# Supplementary material for: Compensation for X-linked Pdha1 silencing by Pdha2 is essential for meiotic double-strand break repair in spermatogenesis
Source: Development. 2025 Aug 7;152(15):dev204683. doi: 10.1242/dev.204683 (PMC12377818; doi:10.1242/dev.204683)
Supplement: Supplementary information [file develop-152-204683-s1.pdf]

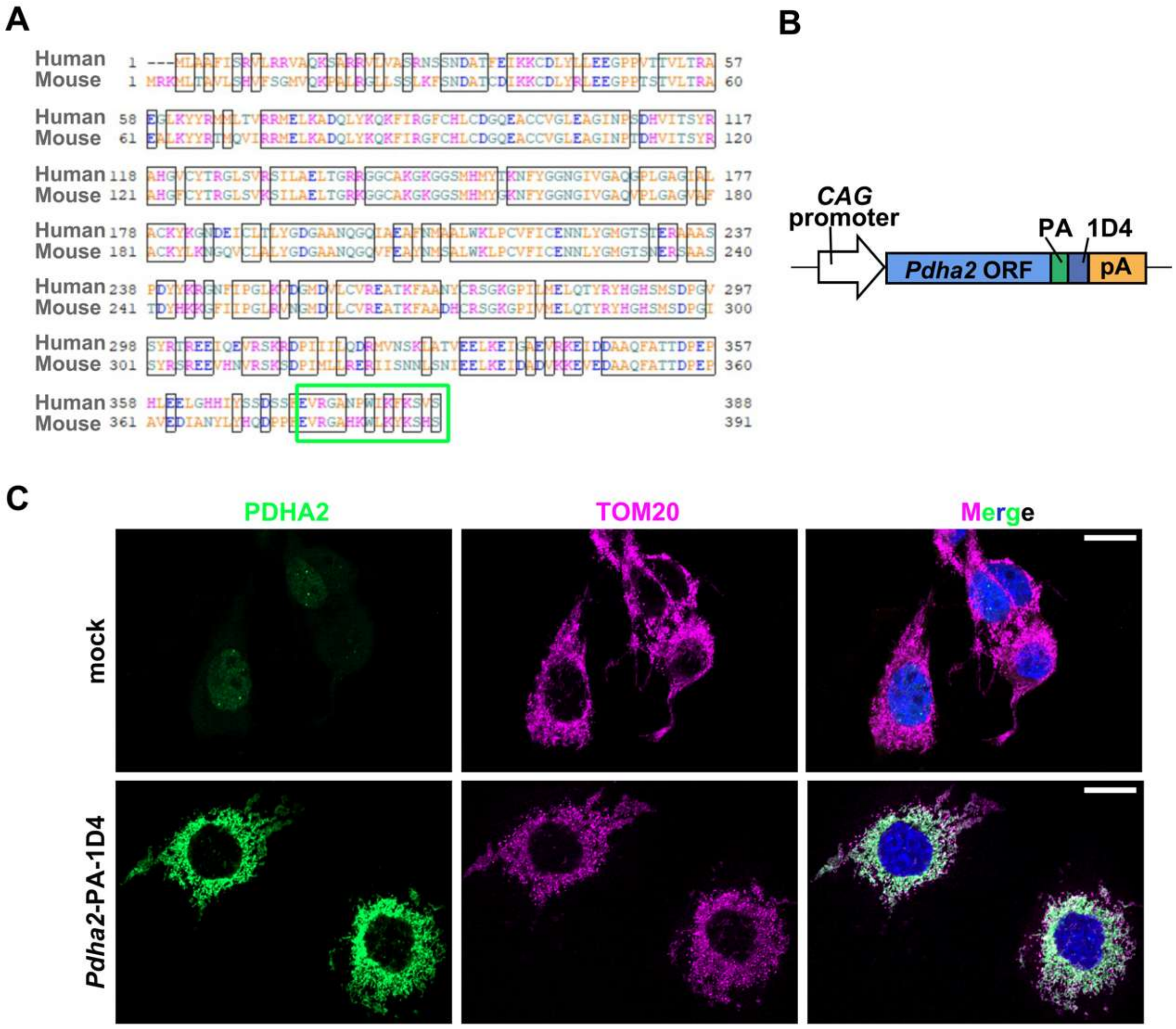

Fig. S1. PDHA2 localizes to mitochondria in cultured cells.

(A) Protein sequence comparison of PDHA2 from human (NP_005381.1) and mouse (NP_032837.1), which shows (358/391) 92% similarity. The green box shows the antibody recognition site of the antibody we used. (B) DNA plasmid structure of *Pdha2* with a PA and 1D4 tag under the CAG promoter. (C) IF analysis of COS-7 cells after plasmid transfection using antibodies against TOM20 (mitochondrial marker, magenta), and PDHA2 (green). Hoechst 33342 (blue) was used for visualizing the nuclei. Scale bars, 20 µm.

Development • Supplementary information

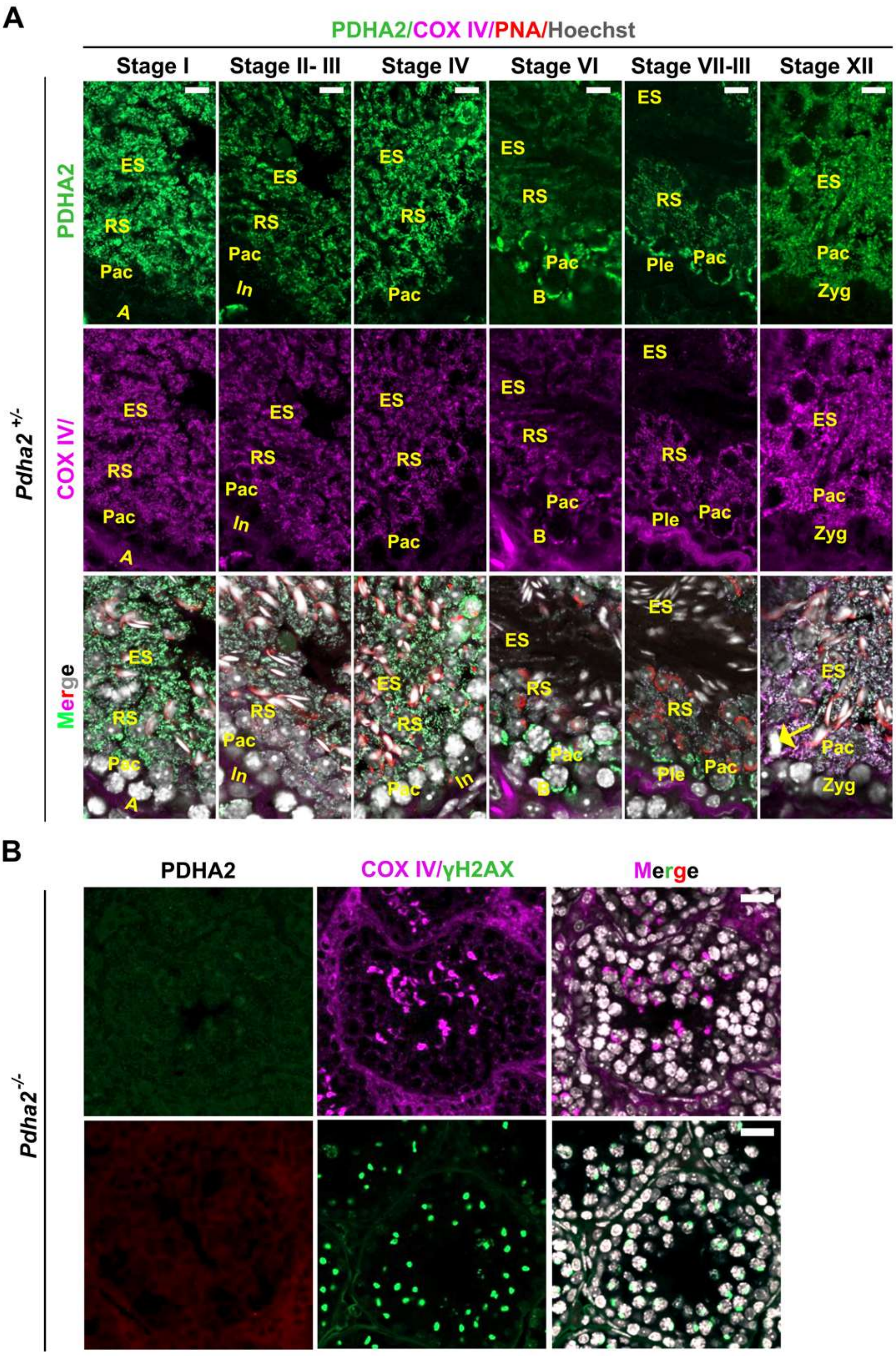

Fig. S2. PDHA2 starts to express in mitochondria at the pachytene stage.
(A) IF analysis of seminiferous tubules in WT mice with antibodies against COX IV (mitochondrial marker, magenta) and PDHA2 (green). Lectin PNA (red) and Hoechst 33342 (white) were used for visualizing the acrosome and nuclei, respectively. A: type A spermatogonia; B: type B spermatogonia; In: intermediate spermatogonia; Ple: pre-leptotene spermatocytes; Zgy: zygotene spermatocytes; Pac: pachytene spermatocytes; RS: round spermatids; ES: elongating (elongated) spermatids; yellow arrow indicates Metaphase II spermatocytes. Scale bars, 10 µm. (B) Representative images of PDHA2 expression in the adult *Pdha2* KO testis. PDHA2 signals were not observed in *Pdha2* KO testis. Spermatocytes in pachytene stage were recognized by γH2AX foci signals. Scale bars, 20 µm.

Development • Supplementary information

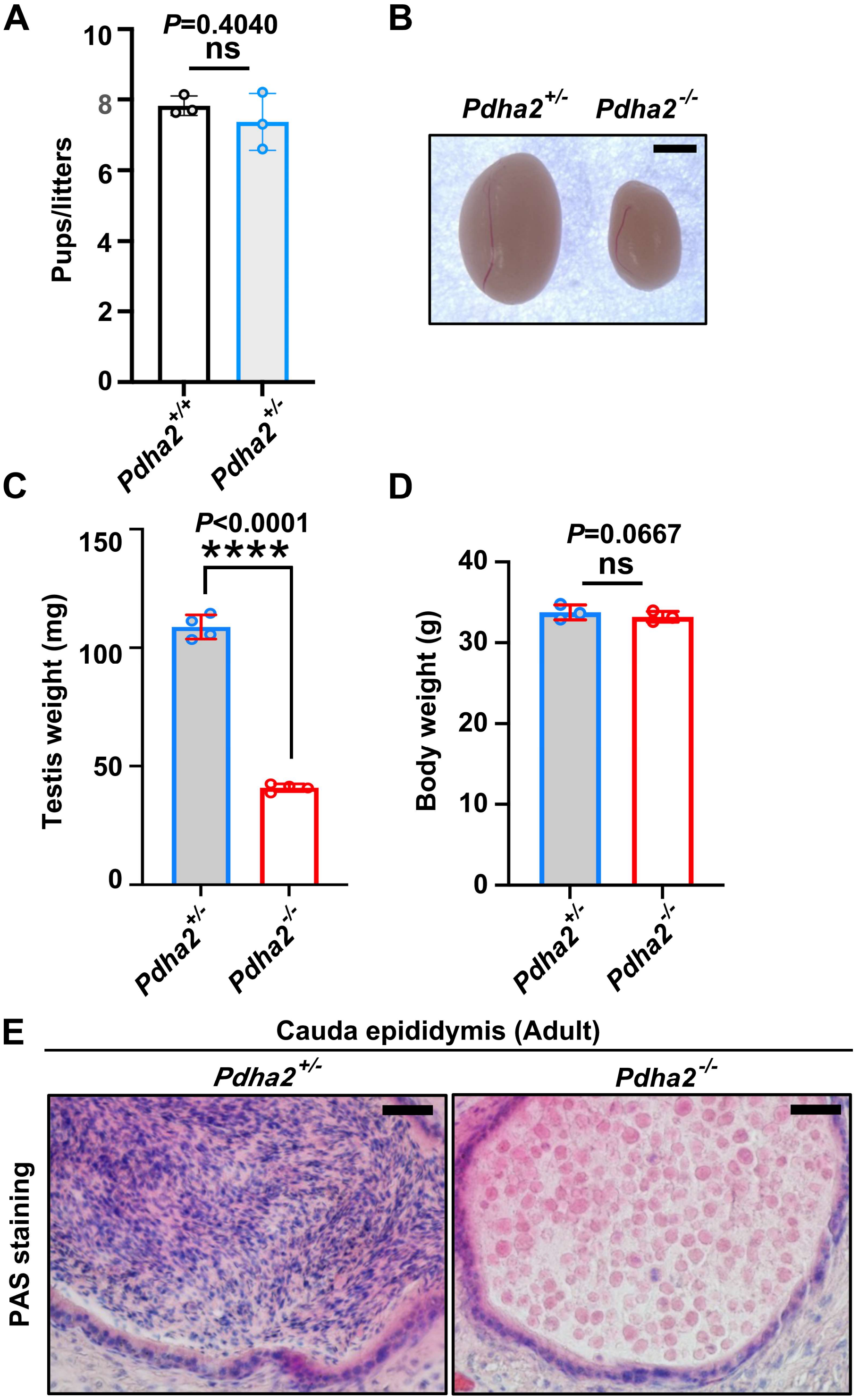
A
P=0.4040
ns
Pups/litters
10
8
6
4
2
0
Pdha2+/+
Pdha2+/-
B
Pdha2+/-
Pdha2-/-
C
P<0.0001
****
Testis weight (mg)
150
100
50
0
Pdha2+/-
Pdha2-/-
D
P=0.0667
ns
Body weight (g)
40
30
20
10
0
Pdha2+/-
Pdha2-/-
E
Cauda epididymis (Adult)
Pdha2+/-
Pdha2-/-
PAS staining

Fig. S3. Histological analysis of *Pdha2* KO male mice.

(A) Number of litters born per plug. Individual adult WT and *Pdha2*$^{+/-}$ male mice (N=3) were mated to three WT female mice. Results from a two-tailed unpaired t-test, ns: no significance. (B) Gross morphology of 12-week-old testis. Scale bar, 2 mm. (C-D) Quantitative analysis of testis (C) and body weight (D) in control and KO mice (N>3). The results of two-tailed unpaired t-tests are indicated in the graphs, ns: no significance. (E) PAS staining of the cauda epididymis of adult mice. No spermatozoa were observed in the cauda epididymis of *Pdha2* KO mice. Instead, abnormal round cells were observed inside the epididymis. Scale bars, 100 µm.

Development • Supplementary information

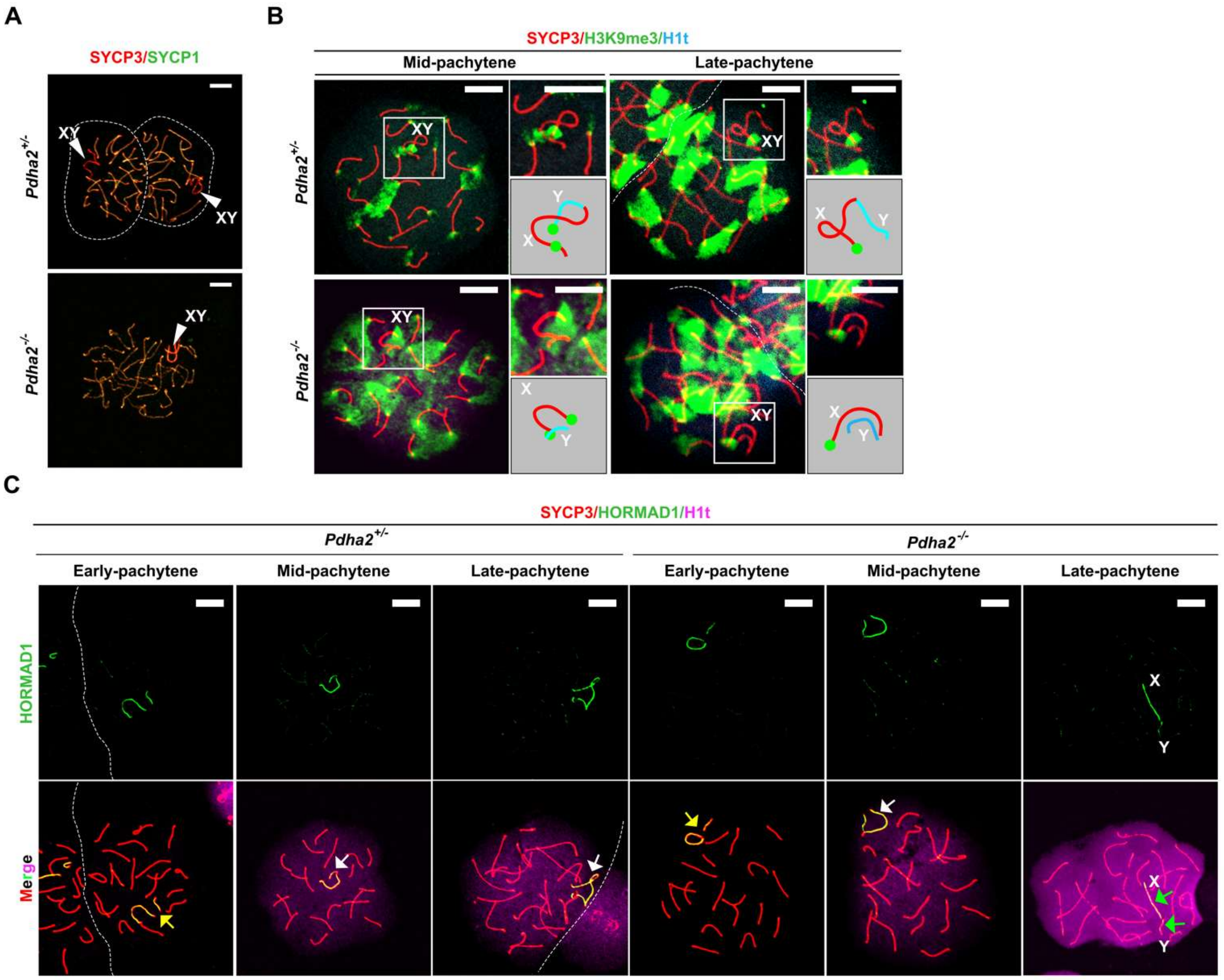

Fig. S4. Premature separation of X and Y chromosomes in *Pdha2* mutant spermatocytes. PND 21 testis were used in these experiments. (A) Representative nuclear spread of spermatocytes obtained from control and *Pdha2* KO testis. Synapsis in pachytene spermatocytes is indicated by SYCP1 localization (green) on chromosomes (SYCP3, red). XY indicates sex chromosomes not stained with SYCP1. Scale bars, 5 µm. (B) H3K9me3 (green) localizes to one end of the autosomes and sex chromosomes in mid-pachytene spermatocytes. The signals on the sex chromosomes express only at one end of the X chromosome at the late pachytene stage. Schematic is shown in the right corner of each image. Chromosomes were stained with SYCP3 (red). H1t (blue) was used to indicate the stages of pachytene spermatocytes. Scale bars, 10 µm. (C) Representative nuclear spreads of pachytene spermatocytes were stained with SYCP3 (red) and HORMAD1 (green, only on the unsynapsed chromosome axes). Yellow arrows represent unpaired sex chromosomes, white arrows indicate the formed sex body, and green arrows show premature separation of the X and Y chromosomes. Scale bars, 5 µm.

Development • Supplementary information

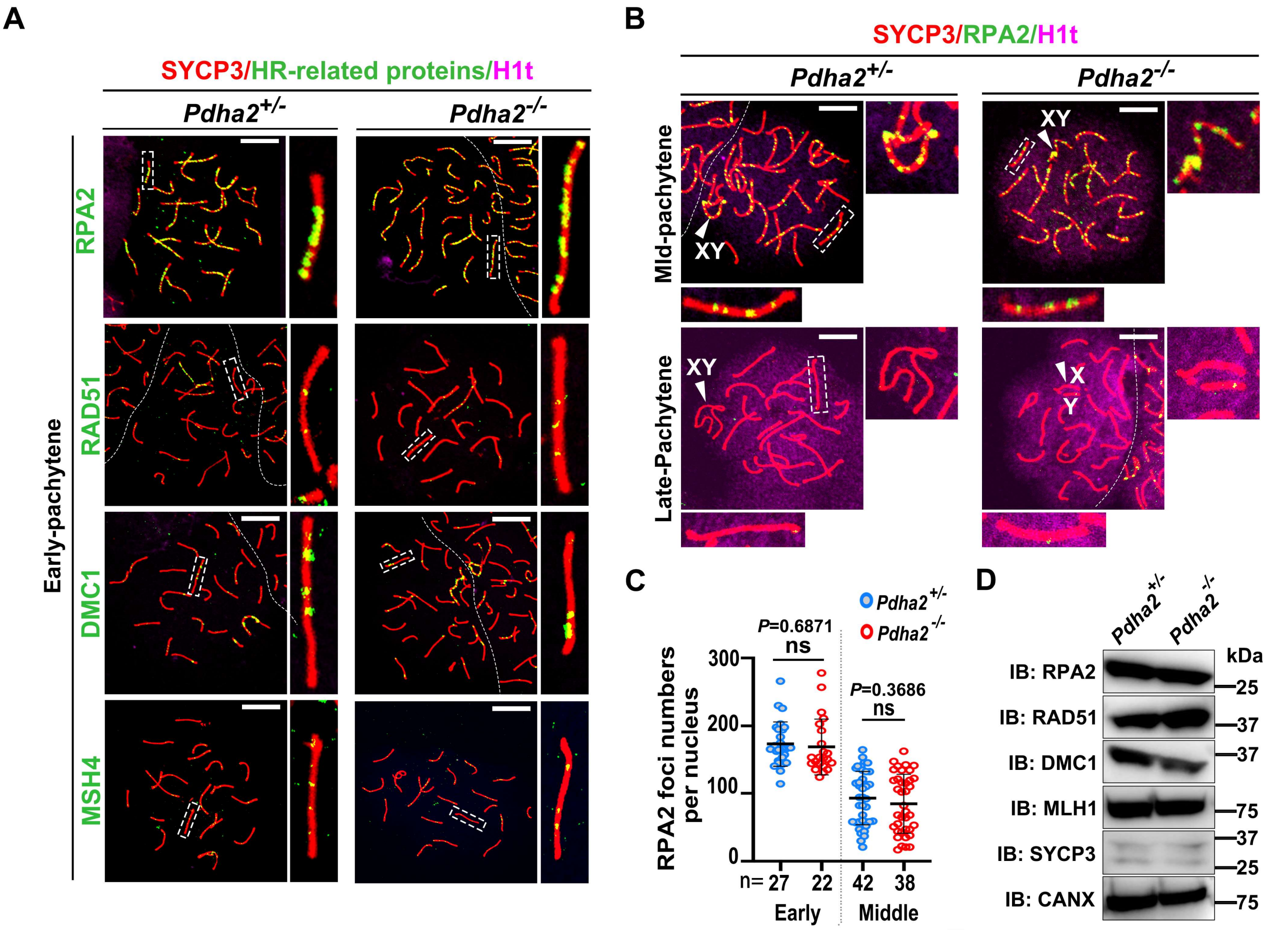

Fig. S5. ***Pdha2*** **KO early-pachytene spermatocytes exhibit normal DSB repair.** PND 21 testis were used in these experiments. (A) Representative nuclear spreads of early pachytene spermatocytes (marked by H1t, magenta) stained for SYCP3 (red) and DSB repair proteins (green) (see Fig. 4A). An autosome is indicated inside the dotted white boxes, and a magnified view is shown on the right. Scale bars, 10 µm. (B) Representative nuclear spreads of middle, and late pachytene spermatocytes (marked by H1t, magenta) stained for SYCP3 (red) and RPA2 (green). Arrowheads indicate the XY body, and magnified views are shown on the right. An autosome is indicated inside the dotted white box, and a magnified view is shown at the bottom. Scale bars, 10 µm. (C) Foci numbers of RPA2 per cell localized on the chromosome axis in the nucleus. *Pdha2*$^{+/-}$ and *Pdha2*$^{-/-}$ spermatocytes at the early/middle-pachytene stages from three mice were scored. The results of two-tailed unpaired t-tests are indicated in the graphs: ns: no significance. (D) WB analysis of DSB repair related proteins (RPA2, RAD51, DMC1, and MLH1) expression in PND 21 mouse testis. The anti-MSH4 antibody was not suitable for WB analysis. SYCP3 and CANX were used as loading controls.

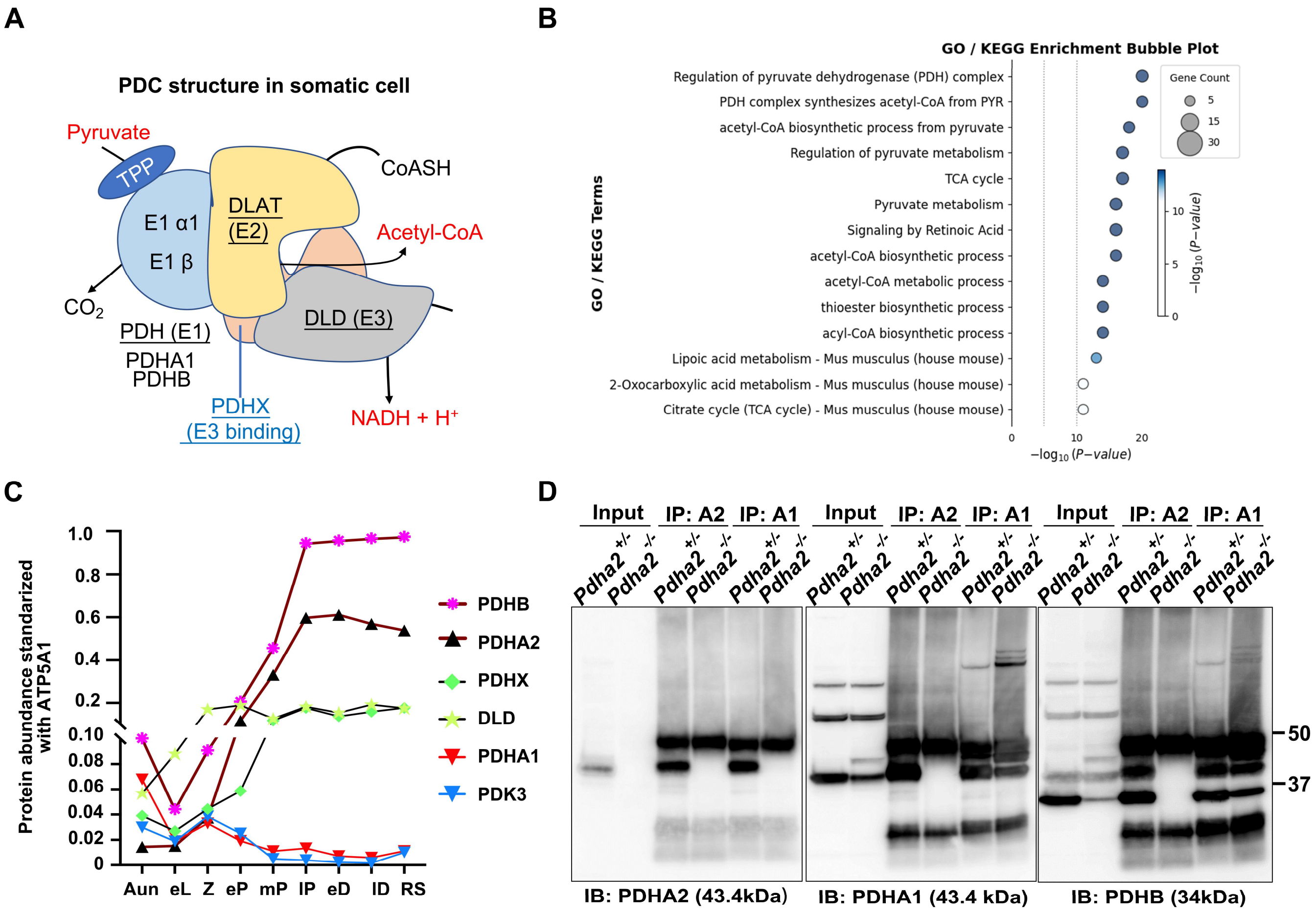

Fig. S6. PDHA2 interacts with proteins that comprise the PDC.

(A) Diagram of the PDC complex in somatic cells. The PDC is comprised of three distinct enzyme activities, pyruvate dehydrogenase (PDH, identified as the E1 component), dihydrolipoamide S-acetyltransferase, (DLAT, identified as the E2 component), and dihydrolipoamide dehydrogenase, (DLD, identified as the E3 component). PDHA1 and PDHB constitute the PDH E1 complex. (B) GO/KEGG analysis with identified proteins by MS analysis after IP with anti-PDHA2 antibody. (C) Protein abundance of PDHA2 and its suggested interacting proteins during mouse spermatogenesis. ATP5A1 was used as a standard mitochondrial protein because of its stable protein expression levels at various stages of spermatogenesis in the testis. Data were collected from published proteome results (Fang et al., 2021). (D) Full-length Western blot images after IP using anti-PDHA1, PDHA2, and PDHB antibodies in adult testis. A2: PDHA2, A1: PDHA1.

Development • Supplementary information

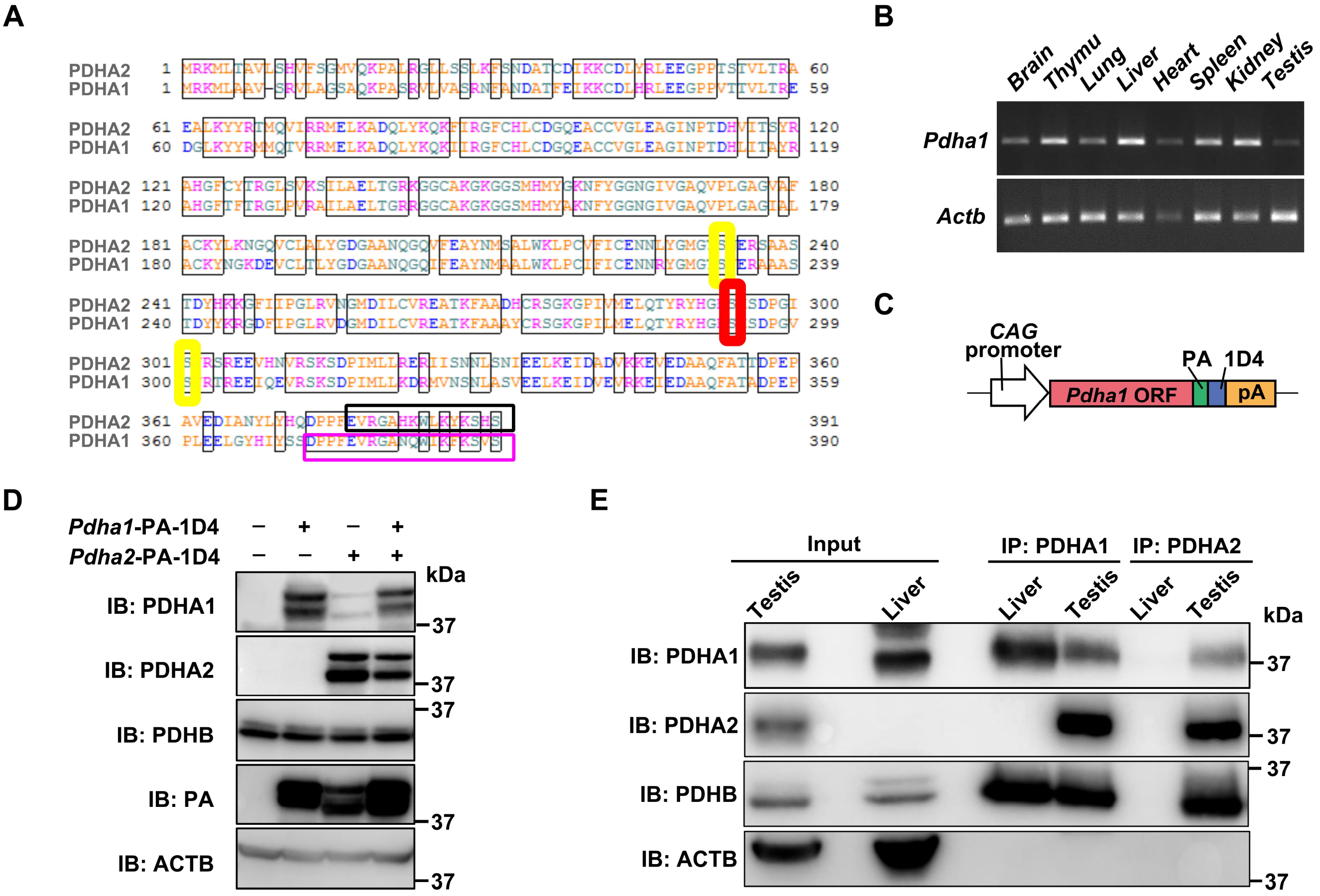

Fig. S7. Validation of anti-PDHA1 and anti-PDHA2 antibodies.

(A) Protein sequence comparison of mouse PDHA2 (NP_032837.1) and PDHA1 (NP_032836.1), demonstrating 95% similarity (373/389). The black and magenta boxes show the antibody recognition sites for the anti-PDHA2 and anti-PDHA1 antibodies we used, respectively. The red box and yellow box show the PDK3 phosphorylation site and predicted phosphorylation site, respectively. (B) RT-PCR for *Pdha1* using cDNA obtained from various mouse tissues. *Atcb* was used as a loading control. (C) Schematic of PA- and 1D4-tagged *Pdha1* under the CAG promoter. (D) WB analysis using anti-PDHA1, PDHA2, and PDHB antibodies. Cell lysates of HEK293T cells transfected with *Pdha1* and/or *Pdha2*-expressing vectors (Fig. S1B, S7C) were examined. PDHB shows strong endogenous signals. ACTB was used as a loading control. (E) WB analyses were performed after IP using anti-PDHA2 or anti-PDHA1 antibodies in the testis and liver samples of adult mice. PDHB was used as it interacts with PDHA1 and PDHA2. ACTB was used as a loading control for input lysates and a negative control for IP experiments.

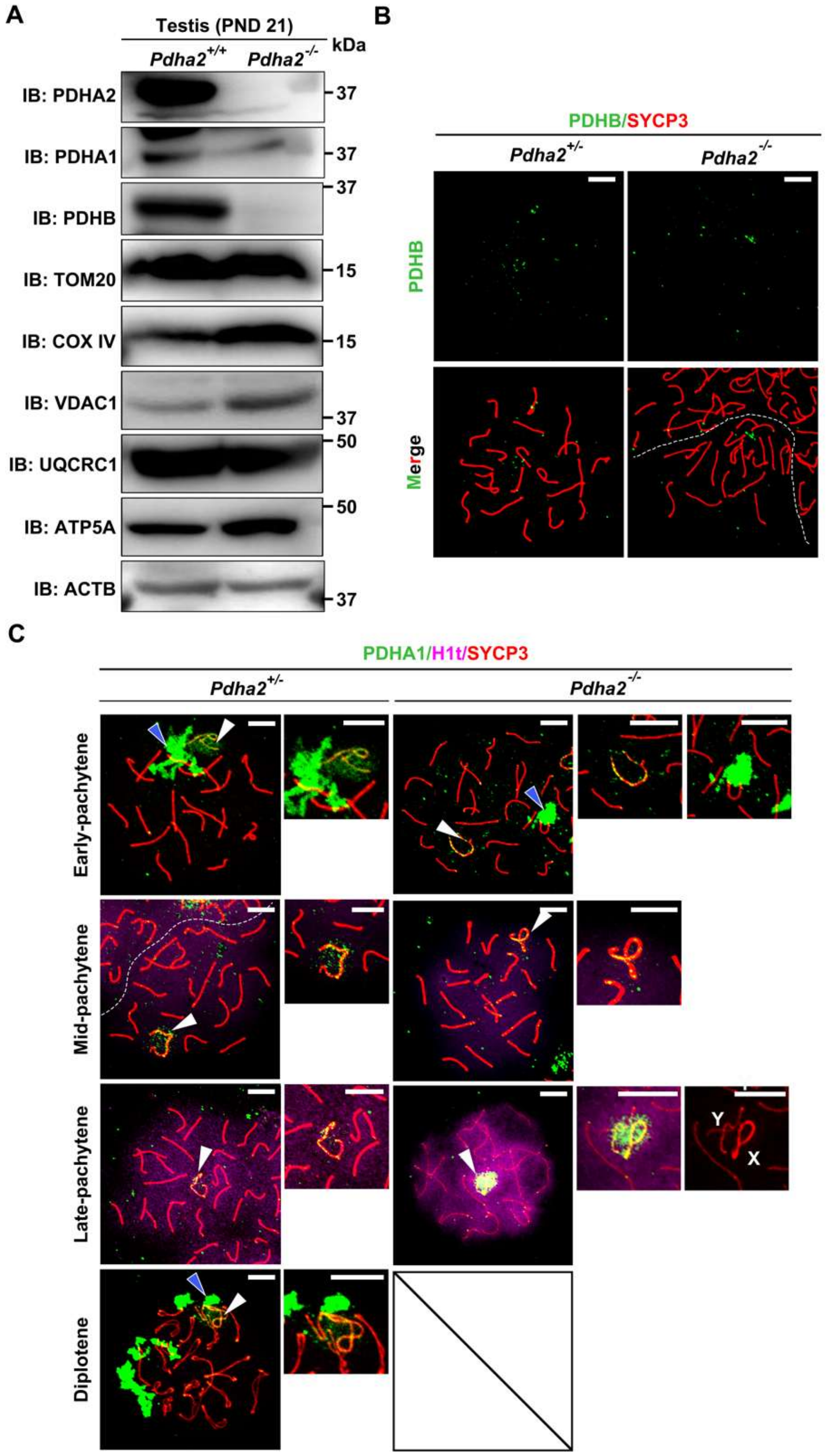

Development • Supplementary information

Fig. S8. Localization of PDHB and PDHA1 in the nucleus.
(A) WB analysis of PDC complex proteins (PDHA2, PDHA1, and PDHB) and mitochondrial proteins (TOM20, COX IV, VDAC1, UQCRC1, and ATP5A) expression in PND 21 mouse testis. ACTB was used as a loading control. (B) Representative nuclear spread using pachytene spermatocytes stained with PDHB (green) and SYCP3 (red) antibodies. PND 21 testis were used. The green debris signal is considered to be the background. Scale bars, 5 μm. (C) Representative nuclear spread using pachytene spermatocytes of PND 21 testis stained with PDHA1 (green), H1t (magenta), and SYCP3 (red) antibodies. Two types of PDHA1 localization, clustered-like granules (blue arrowheads) and XY bodies (white arrowheads), were observed. Scale bars, 5 μm.

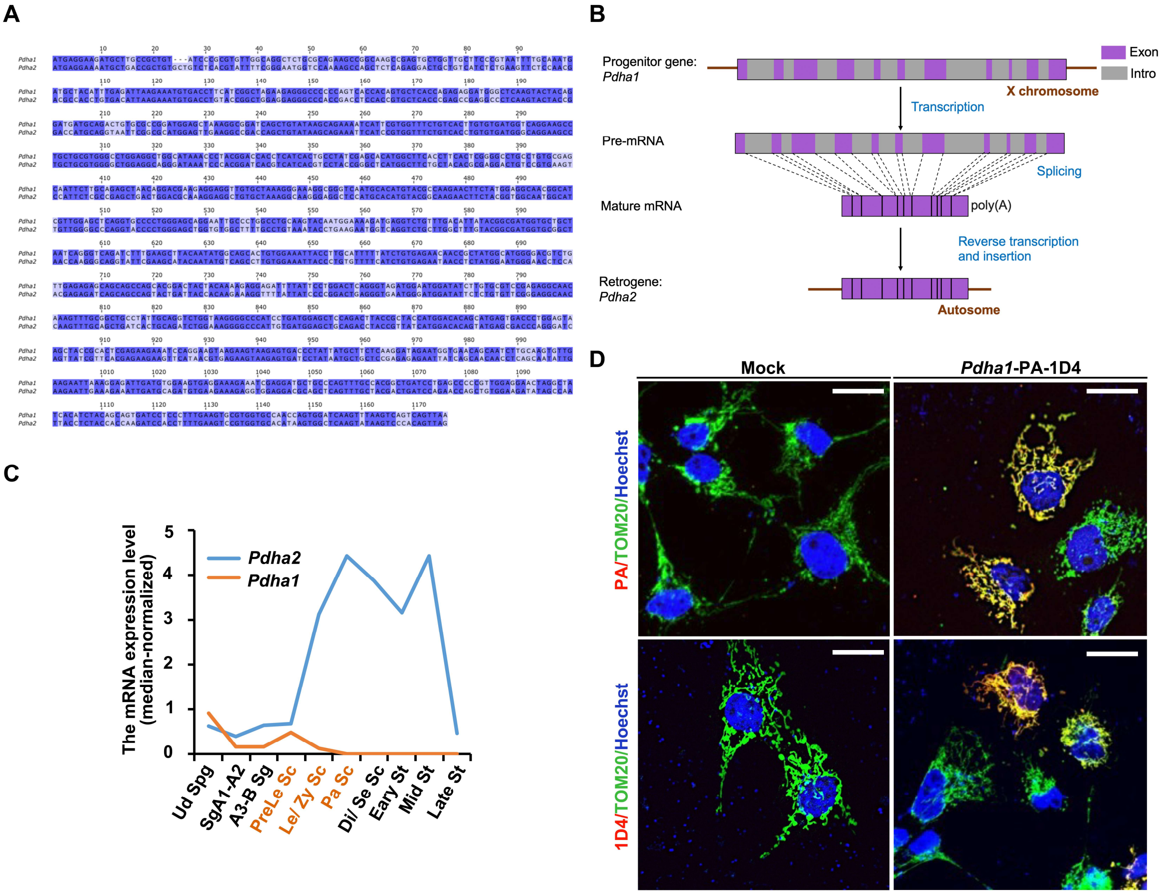

Fig. S9. *Pdha1* is estimated as the progenitor gene for *Pdha2*.

(A) Coding sequence alignment between mouse *Pdha1* and *Pdha2*. (B) Schematic diagram of retrogene formation. The progenitor gene *Pdha1* with 10 introns is illustrated above. The pre-mRNA transcribed from *Pdha1* is spliced and processed into a mature mRNA transcript, which in turn is reverse transcribed and inserted into the autosome, resulting in *Pdha2* (retrogene). Purple bar, exon; gray bar, intron. (C) The mRNA expression patterns of *Pdha1* and *Pdha2* during spermatogenesis. Median-normalized levels are shown. (D) IF analysis of COS-7 cells after transfection with expression vectors. The cells were stained with antibodies against TOM20 (mitochondria marker, green), PA/1D4 (PDHA1, red). Hoechst 33342 (blue) was used for visualizing the nuclei. Scale bars, 20 µm.

Table S1. Primers list

Primer sequence

Primers	Sequence (5'-3')	Figure
Pdha2 Fw (rtPCR)	tccgtgaagtccattctcgc	Fig. 1
Pdha2 Rv (rtPCR)	tgctgatctctcgttggagg	Fig. 1
Pdha2 outer Fw1	aaaacaaccagaggaatggaagg	Fig. 2
Pdha2 outer Rv1	gagcatctctccaagtccct	Fig. 2
Pdha2 Inner Fw2	gctctcagaggactgctgtc	Fig. 2
Pdha2 Inner RV2	catcgccgtacaaagccaag	Fig. 2
Pdha2 Tg Fw3	gcaggacaggtgtgtagacc	Fig. 6
Pdha2 Tg Rv3	gccctcccatatgtccttcc	Fig. 6
Actb rtPCR Fw	ttctacaatgagctgcgtgtggcccc	Fig. 1
Actb rtPCR Rv	gtggtacgaccagaggcatacagggac	Fig. 1
Pdha2-cloning-primer(FW)	aagtcgaccgccgccatgaggaaatgctgaccgc	-
Pdha2 cloning-primer(Rv)	aagaattcactgtgggacttatacttgag	-
Phda1-cloning-primer(FV)	aagtcgacgccgccatgaggaagatgcttgccgc	-
Phda1-cloning-primer(RV)	aactgcagactgactgacttaaacttgatcc	-

Table S2. Antibody information

Antibodies	Source	Identifier	Dillution used in immunofluorescence analysis	Dillution used in immunoblot analysis
Rabbit polyclonal anti-PDHA2	This study		1: 250	1: 500
Rabbit polyclonal anti-PDHA1	This study		1: 500	1: 1000
Rabbit polyclonal anti-PDHB	This study		1: 500	1: 1000
Mouse monoclonal anti-γH2A.X	Millipore	Cat# JBW301	1: 1000	
Mouse monoclonal anti-SYCP3	Santa Cruz Biotechnology	Cat# sc-74569	1: 250	1:500
Rabbit polyclonal anti-SYCP3	Abcam	Cat# ab15093	1: 500	1: 1000
Rabbit polyclonal anti-SYCP1	Abcam	Cat# ab15090	1: 500	
Rabbit polyclonal anti-BRCA1	Generous gift from Dr. Namekawa [1]		1: 500	
Guinea pig anti-H1T	Gift form Dr. M.A. Handel and Dr. E. Marcon [2]		1: 100	
Rabbit polyclonal anti-HORMAD1	ProteinTech	Cat# 13917-1-AP	1: 100	
Rabbit polyclonal anti-RAD51	Abcam	Cat# ab133534	1: 100	1: 500
Rabbit polyclonal anti-DMC1	Santa Cruz Biotechnology	Cat# SC-22768	1: 200	1: 2000
Rabbit polyclonal anti-MSH4	Abcam	Cat# ab58666	1: 100	
Mouse monoclonal anti-MLH1	BD Biosciences	Cat# 551092	1: 100	1: 200
Mouse polyclonal RNA polymerase II *clone CTD4H8	Millipore	Cat# 05-623	1: 2000	
Rabbit polyclonal anti-H3K9me3	Abcam	Cat# ab8898	1: 200	
Rabbit polyclonal anti-RPA2	Abcam	Cat# ab76420	1: 200	1: 500
Mouse monoclonal anti-COX IV	Abcam	Cat# ab33985	1: 250	1: 500
Rabbit polyclonal anti-Tom20 (FL-145)	Santa Cruz Biotechnology	Cat# sc-11415		1: 1000
Mouse polyclonal anti-Tom20 (F-10)	Santa Cruz Biotechnology	Cat# sc-17764	1: 500	
Rabbit polyclonal anti-UQCRC1	ProteinTech	Cat# 21705-1-AP		1: 1000
Mouse monoclonal anti-ATP5A	Santa Cruz Biotechnology	Cat# sc-136178		1: 200
Rabbit polyclonal anti-VDAC1	ProteinTech	Cat# 55259-1-AP		1: 1000
Mouse monoclonal anti-GAPDH	Millipore	Cat# JBW301		1: 1000
Mouse monoclonal anti-β-ACTIN	Abcam	Cat# ab6276		1: 5000
Rabbit polyclonal anti-CALNEXIN	In house	Cat# 99052		1: 1000
Mouse monoclonal anti-1D4 tag	Gift from Dr. Martin M. Matzuk [3]		1: 5000	1: 5000
Rat monoclonal anti-PA	FUJIFILM Wako Chemicals	Cat# 012-25863	1: 1000	1: 1000
Goat anti-rabbit IgG-Alexa Fluor 488	Thermo Fisher Scientific	Cat# A11070	1: 250	
Goat anti-rabbit IgG-Alexa Fluor 546	Thermo Fisher Scientific	Cat# A11071	1: 250	
Goat anti-rabbit IgG-Alexa Fluor 647	Thermo Fisher Scientific	Cat# A21245	1: 250	
Goat anti-rat IgG-Alexa Fluor 488	Thermo Fisher Scientific	Cat# A11006	1: 250	
Goat anti-mouse IgG-Alexa Fluor 488	Thermo Fisher Scientific	Cat# A11017	1: 250	
Goat anti-mouse IgG-Alexa Fluor 546	Thermo Fisher Scientific	Cat# A11018	1: 250	
Goat anti-mouse IgG-Alexa Fluor 647	Thermo Fisher Scientific	Cat# A21235	1: 250	
Goat anti-guinea pig IgG-DyLight 350	Thermo Fisher Scientific	Cat# SA5-10093	1: 250	
Goat anti-guinea pig IgG-Alexa Fluor 488	Thermo Fisher Scientific	Cat# A11073	1: 250	
Goat anti-guinea pig IgG-Alexa Fluor 546	Thermo Fisher Scientific	Cat# A11074	1: 250	
Goat anti-guinea pig IgG-Alexa Fluor 647	Thermo Fisher Scientific	Cat# # A-21450	1: 250	
Goat anti-rabbit IgG-HRP	Jackson ImmunoResearch	Cat# 111-036-045		1: 2000
Goat anti-rat IgG-HRP	Jackson ImmunoResearch	Cat# 112-035-167		1: 10000
Goat anti-mouse IgG-HRP	Jackson ImmunoResearch	Cat# 115-036-062		1: 5000

[1] Ichijima Y, Ichijima M, Lou Z, Nussenzweig A, Camerini-Otero RD, Chen J et al. MDC1 directs chromosome-wide silencing of the sex chromosomes in male germ cells. Genes Dev. 2011; 25(9):959-71. doi: 10.1101/gad.2030811. PubMed PMID: 21536735.

[2] Inselman A, Eaker S, Handel MA (2003) Temporal expression of cell cycle-related proteins during spermatogenesis: establishing a timeline for onset of the meiotic divisions. Cytogenet Genome Res 103: 277–284.

[3] Ishiguro KI, Matsuura K, Tani N, Takeda N, Usuki S, Yamane M *et al*. MEIOSIN Directs the Switch from Mitosis to Meiosis in Mammalian Germ Cells. Dev Cell. 2020 Feb 24;52(4):429-445.e10. doi: 10.1016/j.devcel.2020.01.010. Epub 2020 Feb 6. PMID: 32032549.
